# A Case of Charcot Spine Arthropathy at the Lumbosacral Level in a Patient With Ankylosis of the Spine

**DOI:** 10.7759/cureus.80656

**Published:** 2025-03-16

**Authors:** Yoshiaki Oda, Koji Uotani, Tomoko Tetsunaga, Kensuke Shinohara, Toshifumi Ozaki

**Affiliations:** 1 Department of Orthopedic Surgery, Okayama University Hospital, Okayama, JPN; 2 Department of Musculoskeletal Traumatology, Okayama University Graduate School of Medicine, Dentistry, and Pharmaceutical Sciences, Okayama University, Okayama, JPN; 3 Department of Orthopedic Surgery, Faculty of Medicine, Dentistry, and Pharmaceutical Sciences, Okayama University, Okayama, JPN

**Keywords:** ankylosing spine, charcot spine, charcot spine arthropathy, lumbosacral segment, paraplegia, transdiscal screw

## Abstract

Charcot spinal arthropathy, a rare refractory progressive disease, is characterized by symptoms such as pain, deformity, and neurological impairment, which can significantly reduce functional ability, quality of life, and life expectancy. We report a case of Charcot spine at the L5/S1 level with long segment ankylosis to the L5 vertebra. We first performed thorough debridement via a posterior approach. We used antibiotic-containing cement as a spacer to fill the dead space, facilitating the second surgery approach. In the second surgery, transdiscal screws, which have a low profile and strong force, were used as anchors, and bulk bone harvested from both iliac bones was grafted to the intervertebral space. The lumbosacral alignment was kyphotic, and the patient could sit and move independently. Disimpaction was impossible, and a stoma had to be created.

## Introduction

Charcot spine arthropathy is a rare, refractory, and progressive pathology [1-7]. The symptoms of Charcot spine arthropathy include pain, deformity, and neurological impairment, which can significantly reduce functional ability, quality of life, and life expectancy [8]. Although conservative and surgical treatments are available, no standard treatment has yet been established. Surgery should be considered on a case-by-case basis. It is reported that posterior surgery alone has a high rate of pseudoarthrosis and implant failure, leading to a high number of reoperations [9]. Most of the surgical procedures currently performed are circumferential spinal fusions involving anterior and posterior constructs.

We report our experience treating a patient with Charcot spine arthropathy at the L5/S1 level who had widespread ankylosis from the thoracic to the lumbar spine.

## Case presentation

A 58-year-old man had a thoracic spinal cord injury due to a traffic accident at age 21. The patient underwent posterior fixation and had left complete paraplegia at neurological level T9. The patient could move around in a wheelchair, change clothes, and wash independently. He controlled his stools using an enema and underwent occasional disimpaction.

The patient developed a fever of 40°C. Four days later, his family doctor prescribed antibiotics; however, his condition did not improve. Six days after fever onset, the patient visited a general hospital. His white blood cell count (WBC) was 16,010/μL, and his C-reactive protein (CRP) level was 33.3 mg/dL. Furthermore, a computed tomography (CT) scan showed destruction of the L5/S1 intervertebral space and fluid retention. He was referred to our hospital's Department of Internal Medicine, where he underwent various culture tests and intravenous infusions of vancomycin (VCM) and cefazolin (CEZ).

**Figure 1:**
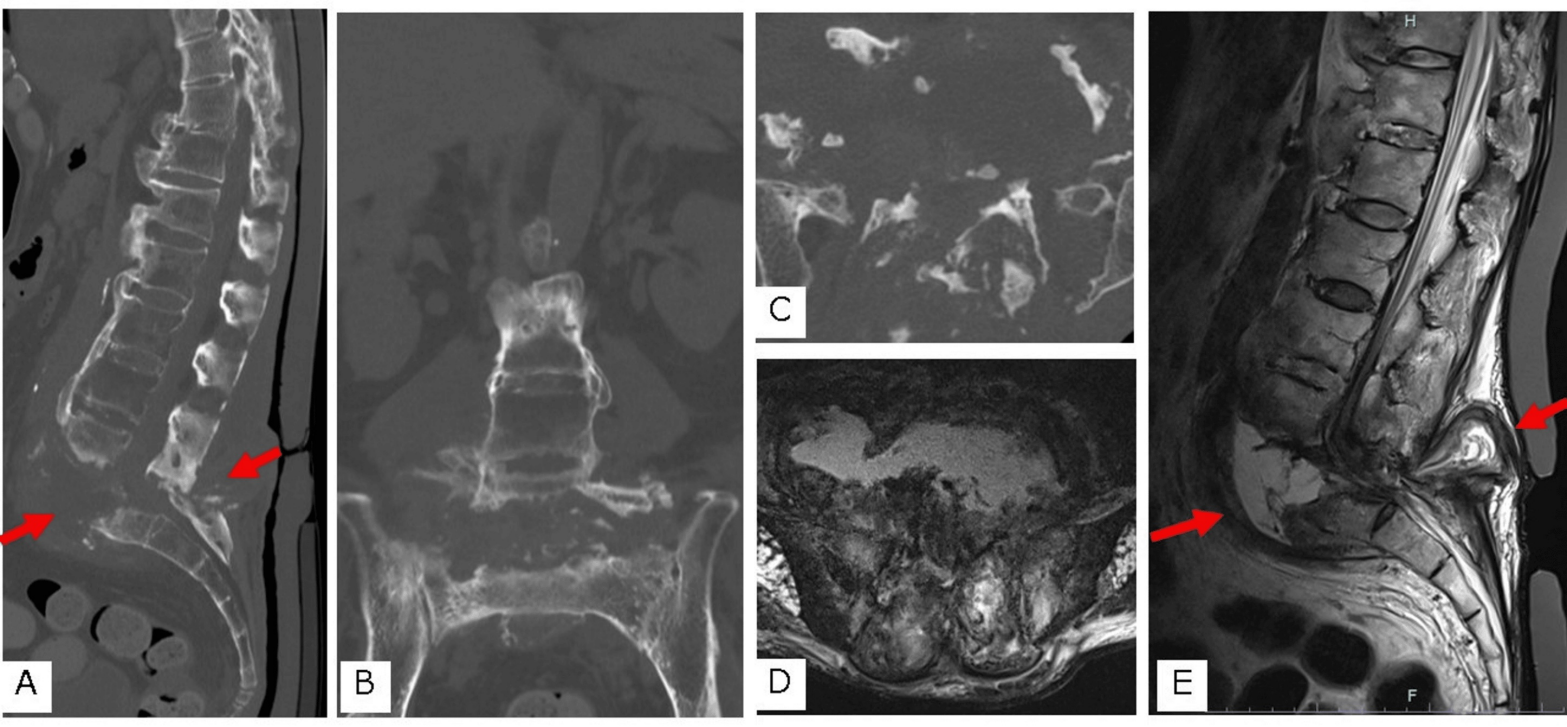
Preoperative computed tomography (CT) and magnetic resonance imaging (MRI) A. Lumbosacral mid-sagittal reconstruction CT; B. Lumbosacral coronal CT; C. Axial image at the L5/S1 level; D. T2-weighted axial image at the L5/S1 level; E. T2-weighted lumbosacral mid-sagittal image

On day 9 after the fever onset, the patient was referred to our department. The CT showed destruction of the L5/S1 intervertebral space and local lordosis, with spinal ankylosis from the proximal thoracic vertebrae to the fifth lumbar vertebra (Figures 1A-1C). Magnetic resonance imaging (MRI) showed that the enlarged L5/S1 intervertebral disc space was filled with fluid passing through the spinal canal and connecting with the posterior soft tissues (Figures 1D, 1E).

The patient did not consent to surgical treatment, and conservative treatment was performed for 2.5 months. The patient's condition did not improve; therefore, we explained the condition to him again and obtained his consent for surgery.

A two-stage procedure was performed. The first operation aimed to perform debridement, collect specimens for diagnosis, and place an antibiotic-containing cement spacer in the dead space. In the second surgery, we planned to perform bone grafting and fixation using a spinal implant. To minimize impairment to the patient's activities of daily living (ADLs), we aimed to achieve kyphotic alignment that would allow catheterization and fecal disimpaction.

**Figure 2:**
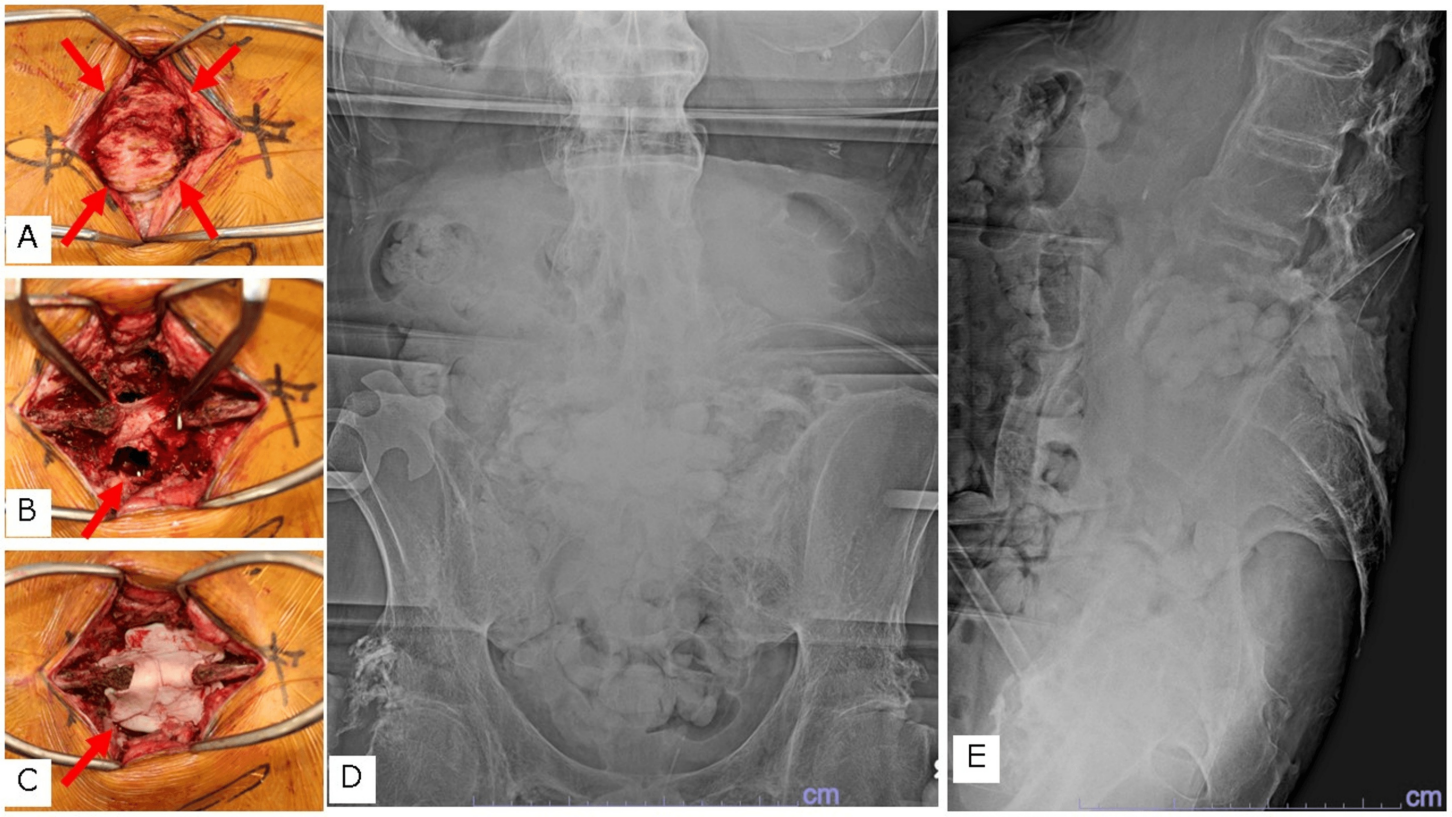
Intraoperative image and postoperative X-ray of the first surgery A. Enbloc resection of the encapsulated inflammatory soft tissues was performed as much as possible. B. Debridement of the posterior soft tissue and intervertebral space was performed. C. A cement spacer was placed in the intervertebral and interlaminar space to provide stability. D. Anteroposterior postoperative X-ray; E. Lateral postoperative X-ray

In the first surgery, a skin incision was made from the L4 to S2 spinous process. The cystic lesion in the muscle layer was excised along the septum (Figure 2A). A lamina spreader was placed between the L5 and S1 laminae, and the soft tissue around the dura was excised. An O-arm image was obtained after a large incision was made in the L5/S1 intervertebral disc (Figure 2B). Debridement was performed using a navigated chisel up to the bony end plate. The debridement area was washed with 6000 ml of saline using a pulsed irrigator and then with iodine saline. A spacer was prepared by mixing 40 g of polymethylmethacrylate (PMMA) with 4 g of VCM and 180 mg of gentamicin (GM). After creating a bead-like shape, the intervertebral space was filled. In the dorsal dead space of the dura, a Penrose drain was placed to prevent thermal damage, and a cement spacer was placed to fill the interlaminar space (Figures 2C-2E). The Penrose drain was removed after hardening).

**Figure 3:**
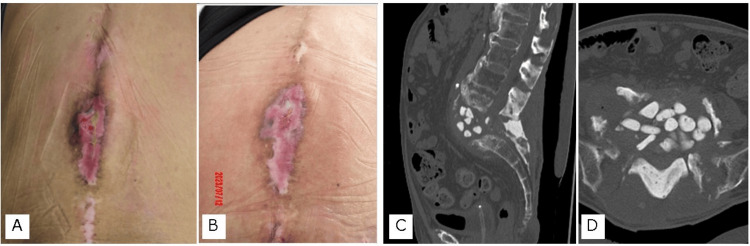
Preoperative bed sore condition and computed tomography (CT) A. Pressure ulcer at the apex of the kyphosis before the first surgery; B. Pressure ulcer at the apex of the kyphosis before the second surgery; C Lumbosacral mid-sagittal reconstruction CT before the second surgery; D. Lumbosacral axial CT at the L5/S1 level before the second surgery

The operative time was 283 minutes, and the intraoperative blood loss was 600 ml. After surgery, the patient was prohibited from lying in a supine position below 30° and only underwent rehabilitation in bed. A long-term culture was also performed; however, the test results were negative. Pathological examination did not reveal neutrophil infiltration, suggesting no infection. Intravenous nutrition was administered to improve the patient's nutritional status, and a second surgery was performed four weeks later. After confirming that the bedsore at the apex of the kyphosis showed signs of improvement, CT was performed before the second surgery (Figure 3).

**Figure 4:**
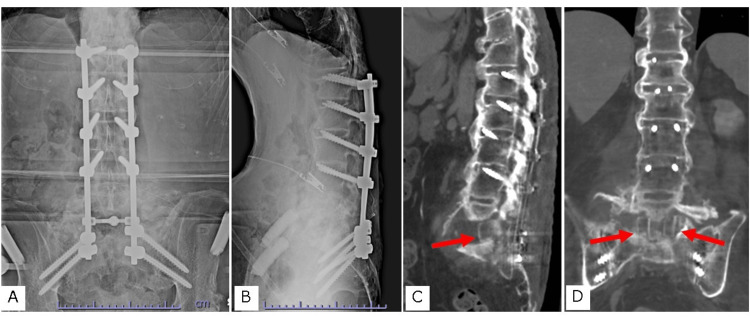
Postoperative X-ray and computed tomography (CT) A. Anteroposterior postoperative X-ray; B. Lateral postoperative X-ray; C. Sagittal plane of lumbosacral CT; D. Coronal plane of CT shows bulk bone grafts

For the second surgery, the patient was placed in the prone position on a Jackson table with the head lowered using a Mayfield clamp and the chest pad elevated to prevent lordosis in the L5/S1 space. First, toricortical bone was harvested from both iliac crests. The skin was incised from the Th12 spinous process to the S2 spinous process, and surgery was initiated. The osteophytes on the dorsal side of the vertebral arch were resected to place the screw as low as possible. Transdiscal screws were placed from L1 to L4 during navigation. The spinous processes were resected such that they were at the same height as the screw heads. The antibiotic-containing spacer placed during the previous surgery made the approach easier and was easy to remove. The harvested bone was formed into four toricortex bones, each approximately 20 mm high, and inserted into the intervertebral space. The 6.0 mm cobalt chrome rods were then tightened, and the alignment was further adjusted to a kyphotic alignment using in situ bending (Figure 4).

After the second surgery, the patient was allowed to rest without restrictions, and rehabilitation was initiated. The wound healed without any complications, and the pressure ulcer improved. The patient was transferred to a rehabilitation hospital six weeks later.

**Figure 5:**
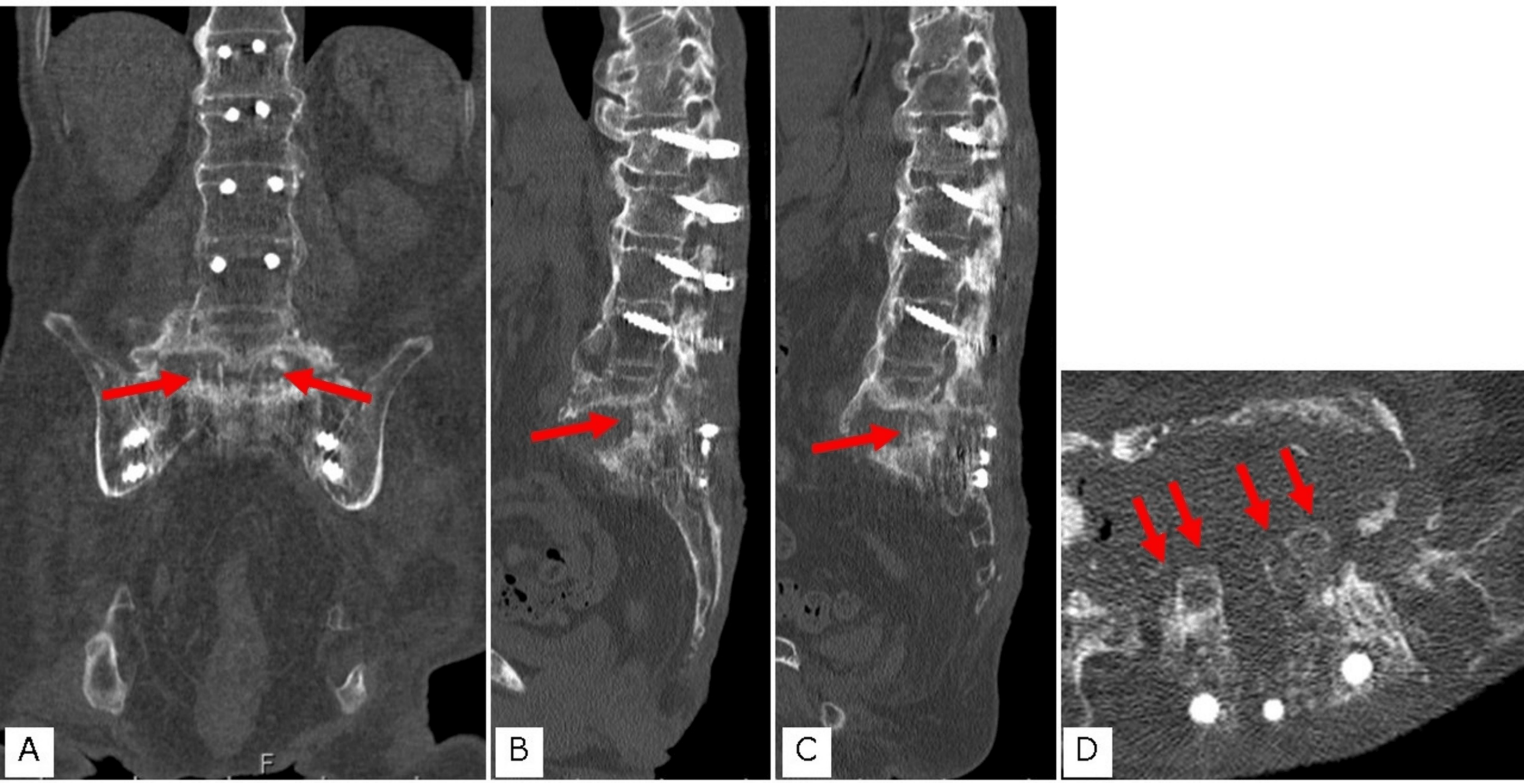
Computed tomography (CT) obtained six months after surgery A. Coronal plane; B and C. Sagittal plane; D. Axial plane at the L5/S1 level. The bone grafted in the intervertebral space and around the rods was not absorbed.

**Figure 6:**
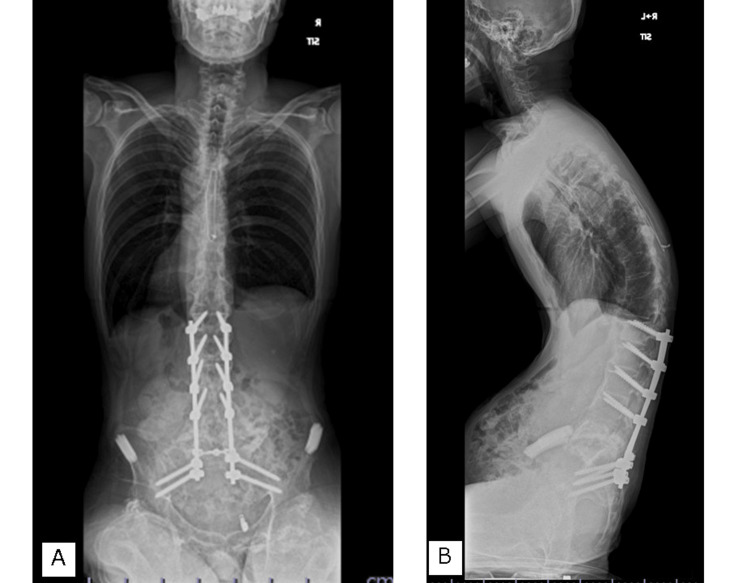
Final follow-up X-ray A. Anteroposterior X-ray with independent sitting; B. Lateral Xray with independent sitting

The patient was discharged after three months. The patient could turn over in bed, sit free with both arms, and be transferred to a wheelchair without any problems. He was able to self-catheterize and wipe his anus after defecation but was unable to insert his finger deep enough into the anus for disimpaction. A stoma was created, and the patient could manage his bowel movements independently and more easily than before. The CT showed bone union six months after surgery (Figure 5), which was maintained one year after surgery (Figure 6).

## Discussion

The best surgical treatment strategy for Charcot spine is yet to be established [10]. The main surgical goals involve debridement of the inflamed tissue, decompression of the spinal canal stenosis, and stabilization with a 360° bone graft [2]. Surgical treatment can be performed as a two-stage procedure using an anterior and posterior approach or as a one-stage procedure using a posterior approach.

In the present case, the L5/S1 segment was destroyed at 360°. Therefore, we decided to perform 360° debridement, including the bone graft bed. The posterior approach was selected because it was necessary to further reduce lordosis, and we planned to insert a bone graft with a height as low as possible. A two-stage operation was planned to improve nutritional status, alleviate local inflammation, and analyze the results of the bacterial and pathological examinations.

In the first stage, antibiotic-containing cement was used as a spacer in the dead space created by debridement, which facilitated the approach for the second stage and contributed to spinal stability during the waiting period.

Reportedly, the rate of construct failure requiring revision surgery was 29.6% in anterior-posterior combined surgery and 58.3% in posterior surgery [9]. We also considered anterior column reconstruction a very important point and implanted four bone grafts into the debrided intervertebral disc space. In addition, to obtain a strong anchor, a trajectory screw penetrating the intervertebral disc [11] and two sacral alar iliac screws were used for fixation. Additionally, the screws penetrating the endplates were positioned deeper than conventional pedicle screws. The lower-profile screw is beneficial, particularly in the case of a pressure ulcer. A temporary titanium alloy rod was used for correction, which was performed using the translation and in situ bending techniques. Final fixation was performed using a 6.0 mm cobalt-chromium rod, which was almost unbent.

Before surgery, the patient could sit, move in a wheelchair, perform fecal disimpaction, and perform independent ADLs. We adjusted the lordotic spinal alignment to kyphosis to maintain the patient's pre-surgery ADLs. As a result, the patient was able to sit up, move in a wheelchair, and perform catheterization; however, he was unable to perform fecal disimpaction. When fixing the upper thoracic spine to the pelvis in a paraplegic patient, a stoma would likely be required to allow the patient to manage bowel movements independently.

## Conclusions

We encountered a case of Charcot spine at the L5/S1 level with long-segment ankylosis of the L5 vertebra. First, a posterior approach with thorough debridement was performed. The cement spacer filled the dead space and facilitated the approach for the second surgery. In the second surgery, a low-profile and strong transdiscal screw was used as an anchor, and the bone harvested from both iliac bones was grafted onto the intervertebral space. The lumbosacral alignment was kyphotic, and the patient was able to sit and move independently; however, disimpaction was impossible, and a stoma had to be created.
